# Plant Foraging Strategies Driven by Distinct Genetic Modules: Cross-Ecosystem Transcriptomics Approach

**DOI:** 10.3389/fpls.2022.903539

**Published:** 2022-07-04

**Authors:** Yusaku Sugimura, Ai Kawahara, Hayato Maruyama, Tatsuhiro Ezawa

**Affiliations:** ^1^Graduate School of Agriculture, Hokkaido University, Sapporo, Japan; ^2^Health & Crop Sciences Research Laboratory, Sumitomo Chemical, Co., Ltd., Takarazuka, Japan

**Keywords:** arbuscular mycorrhiza, field transcriptomics, gene coexpression network, maize, foraging strategies

## Abstract

Plants have evolved diverse strategies for foraging, e.g., mycorrhizae, modification of root system architecture, and secretion of phosphatase. Despite extensive molecular/physiological studies on individual strategies under laboratory/greenhouse conditions, there is little information about how plants orchestrate these strategies in the field. We hypothesized that individual strategies are independently driven by corresponding genetic modules in response to deficiency/unbalance in nutrients. Roots colonized by mycorrhizal fungi, leaves, and root-zone soils were collected from 251 maize plants grown across the United States Corn Belt and Japan, which provided a large gradient of soil characteristics/agricultural practice and thus gene expression for foraging. RNA was extracted from the roots, sequenced, and subjected to gene coexpression network analysis. Nineteen genetic modules were defined and functionally characterized, from which three genetic modules, mycorrhiza formation, phosphate starvation response (PSR), and root development, were selected as those directly involved in foraging. The mycorrhizal module consists of genes responsible for mycorrhiza formation and was upregulated by both phosphorus and nitrogen deficiencies. The PSR module that consists of genes encoding phosphate transporter, secreted acid phosphatase, and enzymes involved in internal-phosphate recycling was regulated independent of the mycorrhizal module and strongly upregulated by phosphorus deficiency relative to nitrogen. The root development module that consists of regulatory genes for root development and cellulose biogenesis was upregulated by phosphorus and nitrogen enrichment. The expression of this module was negatively correlated with that of the mycorrhizal module, suggesting that root development is intrinsically an opposite strategy of mycorrhizae. Our approach provides new insights into understanding plant foraging strategies in complex environments at the molecular level.

## Introduction

Plants have evolved diverse strategies for the acquisition of mineral nutrients, in particular nitrogen (N) and phosphorus (P). Approximately 400 Mya, early plants without a functional root system associated with arbuscular mycorrhizal (AM) fungi to acquire water and nutrients (Simon et al., 1993; Remy et al., 1994; Redecker et al., 2000; Humphreys et al., 2010). This assisted plant terrestrialization and thus is the most ancient strategy of land plants for root foraging. Fungi are associated with more than 70% of modern land plants (Brundrett and Tedersoo, 2018) and construct hyphal networks in soil, facilitating an extensive surface area for water/nutrient acquisition, that is, the mycorrhizal pathway (Smith and Read, 2008). P and N deficiencies trigger the secretion of plant hormone strigolactones into the rhizosphere (Yoneyama et al., 2012), which promotes contact of fungal hyphae with roots by stimulating hyphal branching (Akiyama et al., 2005). After physical contact, fungal hyphae penetrate into the cortex and form highly branched hyphal termini “arbuscules” where nutrient exchange occurs between symbionts. Briefly, inorganic phosphate (Pi), nitrate (NO_3_^–^), and ammonium (NH_4_^+^) are taken from the soil by extraradical hyphae, delivered to the arbuscules, and released into the arbuscular interface (reviewed in Ezawa and Saito, 2018) from which plant cells take nutrients *via* mycorrhiza-specific transporters for Pi (Rausch et al., 2001; Harrison et al., 2002), NO_3_^–^ (Wang et al., 2020), and NH_4_^+^ (Koegel et al., 2013). In return, the host supplies organic carbon as carbon source for fungi (reviewed in Salmeron-Santiago et al., 2022). Briefly, lipids (Bravo et al., 2017; Jiang et al., 2017; Keymer et al., 2017; Luginbuehl et al., 2017) and sugars (Helber et al., 2011) are exported *via* the putative lipid exporter (i.e., a complex of the half-sized ABC transporters STR and STR2) (Zhang et al., 2010) and members of the sugar transporter SWEET gene family (An et al., 2019), respectively.

The morphological plasticity of roots is an important part of plant foraging strategies given that roots provide another major pathway for nutrient uptake, that is, the root-direct pathway. NO_3_^–^- and NH_4_^+^-enriched patches induce the localized proliferation of lateral roots for efficient capture of nutrients (Drew, 1975), which is triggered by NO_3_^–^ and NH_4_^+^ uptake *via* the plasma membrane NITRATE TRANSPORTER 1 (NRT1) (Remans et al., 2006; Ho et al., 2009) and AMMONIUM TRANSPORTER (AMT) (Lima et al., 2010), respectively. Lateral root formation is, however, inhibited under severe N deficiency, because saving carbon in resource-limited environments is an essential trait for survival (Araya et al., 2014). Pi patches also promote localized proliferation of lateral roots (Drew, 1975). P deficiency generally inhibits primary root growth but increases lateral root growth and density, leading to a shallow root system (Williamson et al., 2001; Gruber et al., 2013). Root hairs also play a significant role in Pi uptake by enlarging the contact surface area for the soil solution (Gahoonia et al., 2001).

Physiological responses for Pi acquisition under low Pi conditions, known as Pi starvation response (PSR), are also important strategies and have extensively been studied. Pi availability in soil is generally low because a large part of P is present as sparingly soluble inorganic salts and organic P that are unavailable for plants (Shen et al., 2011). Under such conditions, i.e., plants upregulate the high-affinity Pi transporter genes of the Pht1 family to enhance root uptake capability, secrete non-specific acid phosphatase and organic acids to increase the soil Pi pool, and replace phospholipids with sulfo- and galactolipids to accelerate internal Pi recycling, which are typical PSRs (reviewed in Plaxton and Tran, 2011).

Despite extensive molecular/physiological studies on individual strategies under controlled laboratory/greenhouse conditions, there is little information about how plants orchestrate these strategies in the field, that is, in complex and changing environments. Plants are likely to upregulate the genes responsible for PSR, mycorrhiza formation, and root development in parallel under P-deficient conditions. It is unknown, however, how plants prioritize (i.e., modulate resource allocation to) these strategies to optimize the overall efficiency under various levels of P deficiency. N deficiency would reduce the relative value of P even at low P availability (Johnson, 2010) and thus downregulate genes for PSR and root development. Genes for mycorrhiza formation, however, may not be downregulated by N deficiency, because both P and N could be acquired through the mycorrhizal pathway. Disentangling such complex gene-environment interactions under field conditions is a great challenge but will contribute not only to understanding the regulatory mechanism of plant foraging strategies but also to sustainable intensification of agriculture, e.g., by improving Pi-use efficiency (Plaxton and Tran, 2011) and mycorrhizal function (Rillig et al., 2016).

Recently, transcriptomics has been applied to several field studies in plant science: temporal/seasonal changes in the transcriptome (Nagano et al., 2012, 2019) and responses to fertilizers (Yu et al., 2018) and drought stress (Varoquaux et al., 2019). Particularly, the application of transcriptomics followed by weighted gene-coexpression network analysis (WGCNA) leads to the identification of genetic modules for, e.g., microbial symbioses (Varoquaux et al., 2019; Wu et al., 2019) and photosynthesis (Varoquaux et al., 2019). Here, we have established a novel approach, cross-ecosystem transcriptomics; transcriptomes of mycorrhizal roots (i.e., dual transcriptomes of roots naturally colonized by AM fungi) are obtained from plants grown in physically, chemically, and biologically diverse environments and subjected to WGCNA; then, module–environment and module–module interplays are analyzed. This will lead to a comprehensive understanding of plant foraging strategies in the context of plant-microbe-environment interactions at the molecular level.

Maize (*Zea mays* L.) is one of the most important cereal crops worldwide, serving as staple food, livestock feed, and industrial raw material. Most importantly, this crop establishes a mutualistic association with AM fungi and is grown across a wide range of environments/ecosystems, which led us to employ maize as the first model for this approach. We collected plant and soil samples across the United States Corn Belt and Japan; the former achieves the world’s highest productivity with the typical high-input agricultural system, whereas agricultural practice varies regionally in the latter because of diversity in climate and edaphic properties. It was expected that this sampling strategy would provide broad environmental gradients that cannot be provided by greenhouse/laboratory experiments. The present study addressed the following two hypotheses; (i) root foraging strategies are driven by corresponding genetic modules that are upregulated in response to nutrient deficiency, in which (ii) modules involved in N acquisition are driven solely by N deficiency, while those for P acquisition depend not only on P status/availability but also on N status/availability.

## Materials and Methods

### Field Sampling and Soil/Plant Analysis

**FIGURE 1:**
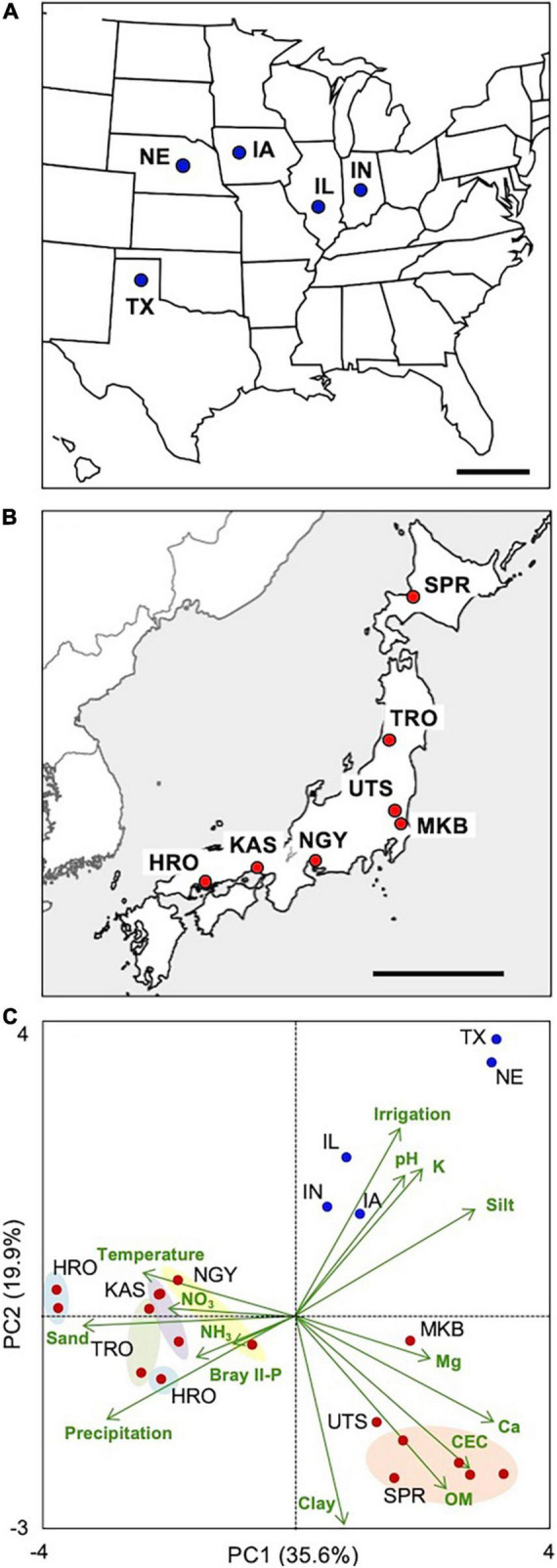
Location and characterization of the maize sampling sites. Roots, shoots, and root-zone soils were collected across the **(A)** United States Corn Belt and **(B)** Japan. **(C)** Principal component analysis of soil, climatic, and agricultural (irrigation) variables of the five sites in the United States and those of 17 experimental plots in the seven sites in Japan. Different experimental plots of the seven sites are circled with colors. Detailed information/data on the plots/sites is provided in Supplementary Tables 1, 4. IL, Illinois; IN, Indiana; IW, Iowa; NE, Nebraska; TX, Texas; SPR, Sapporo; TRO, Tsuruoka; UTS, Utsunomiya; MKB, Makabe; NGY, Nagoya; KAS, Kasai; HRO, Hiroshima. Scale bars: 500 km.

Roots, shoots, and root-zone soils were collected from 13- to 54-day-old maize plants, including 11 genotypes, grown in five field sites across the United States Corn Belt in 2017 (66 plants) and 17 experimental plots in seven field sites across Japan in 2018 (185 plants) (Figures 1A,B). Details of geographic, climatic, and field-management data of the sampling plots/sites are presented in Supplementary Table 1. Leaf number and stem diameter (1–2 cm-above the ground) were measured prior to sampling, and then the root system was collected from a 30 × 30 cm area at a depth of 20 cm and immediately washed with water in a bucket. Primary, lateral, seminal, and crown roots (except for nodal roots) were collected with scissors, cut into small pieces (<1 cm), randomized in water, collected on a stainless mesh, and blotted on a paper towel; about 100 mg of the root pieces were immersed in 15 ml of RNA*later* solution (Thermo Fisher Scientific, Tokyo, Japan). All the processes were completed within 3 min for each sample in the field sites. The aboveground part of the same individuals was collected to measure dry weight, and fully expanded-youngest leaves were subjected to P and N analysis. Approximately 500 g of root-zone soils were collected from the same individuals, air-dried, and sieved for analysis of physical and chemical properties. In some plots/sites, the soil samples from different individuals were combined and mixed, and representative subsamples were analyzed. The root samples in RNA*later* were left for at least 48 h at room temperature, transferred onto a stainless mesh to remove excess RNA*later*, blotted, and stored at −20°C (for temporary storage) or at −80°C (for long-term storage).

Chemical and physical properties of the United States and Japanese soil samples were analyzed at Midwest Laboratories (Omaha, NE, United States) and Tokachi Federation of Agricultural Cooperatives (Obihiro, Japan), respectively, with standard methods. The aboveground part and leaf samples were dried at 80°C for up to 5 days and weighed. The leaf samples were ground and digested with concentrated sulfuric acid and hydrogen peroxide at 200°C for 150 min, and N and P concentrations were determined with the indophenol-blue method (Novamsky et al., 1974) and the ascorbic acid-molybdate blue method (Watanabe and Olsen, 1965), respectively.

### RNA Sequencing and Gene Expression Profiling

The root samples were frozen in liquid nitrogen and ground with a metal cone by Multi-Beads Shocker (Yasui Kikai, Osaka, Japan), and total RNA was extracted using the Maxwell RSC Plant RNA Kit with Maxwell RSC Instrument (Promega, Tokyo, Japan). Sequencing libraries were constructed using KAPA Stranded mRNA-Seq Kit (KAPA Biosystems, Potters Bar, United Kingdom), and single-end 75-base sequencing was performed on an Illumina NextSeq 500 platform (>10 M reads per sample) at Bioengineering Lab (Atsugi, Japan) (mRNA-Seq). In the ribosomal RNA sequencing (rRNA-Seq), libraries were constructed with total RNA without mRNA purification and sequenced on the same platform. Sequence reads with Phred quality score ≥ 20 in >80% of the bases were mapped to the maize B73 reference genome sequence (Zm-B73-REFERENCE-GRAMENE-4.0)^1^ using the HISAT2 program (Kim et al., 2019). Mapped reads were extracted and converted into BAM format using SAMtools (Li et al., 2009), and uniquely mapped reads to protein-coding transcript sequences were counted with the featureCounts program (Liao et al., 2013). Raw read counts were normalized to transcripts per kilobase million (TPM), and then genes with an average TPM ≥ 5 were subjected to subsequent analyses.

To identify maize genes involved in mycorrhiza formation and functioning, Blastp searches were performed against the maize genome using the amino acid sequences of those identified in *Oryza sativa*, *Sorghum bicolor*, *Medicago truncatula*, and *Lotus japonicus* as queries, and candidate genes were subjected to phylogenetic analysis based on the neighbor-joining method with MEGA X (Kumar et al., 2018).

For WGCNA, the TPM data were transformed to logarithmic values (log_2_) and analyzed using the R package WGCNA with the settings of “automatic, one-step network construction,” mergeCutHeight of 0.2, and minimal module size of 30 genes. After the definition of modules, we merged the modules with a distance threshold value of 0.3. The expression levels of modules in individual samples were represented by eigengenes that are principal component 1 (PC1) sample scores calculated based on the expression data of the module member genes (Langfelder and Horvath, 2008). A k-means clustering analysis was performed using correlation distance as a measure with the R package amap. Fisher’s exact test was employed to analyze GO term enrichment using the Blast2GO program with false discovery rates of less than 0.01 and 0.05 for the modules and submodules, respectively (Conesa et al., 2005). The GO annotation list was obtained from the maize genome database.

### rRNA Read Assignment and *de novo* Assembly of Arbuscular Mycorrhizal Fungal mRNA Reads

Maize and AM fungal rRNA sequence reads in the mRNA-Seq data and those in the corresponding rRNA-Seq data were assigned to the maize large-subunit (LSU) rRNA sequence (XR_002749536) and 524 operational taxonomic units (OTUs) of AM fungal LSU rRNA sequences (Niwa et al., 2018) by Blastn searches with the following parameters: similarity, ≥95%; minimum alignment length, ≥75 bp; *E*-value, ≤1e-30. The consensus sequences GTGAAATTGTTGAAAGGGAAACG and GACGTAATGGCTTTAAACGAC at the 5′ and 3′ ends, respectively, of the AM fungal OTU sequences were removed before Blastn. The numbers of the reads assigned to the plant rRNA and AM fungal OTUs were normalized to unit nt length, and total AM fungal read counts in the samples were standardized per 10^5^ plant rRNA reads and transformed to logarithmic values. Non-metric multidimensional scaling (NMDS) was performed for comparing communities revealed by mRNA-Seq and rRNA-Seq with the vegan package^2^ using the Bray–Curtis dissimilarity index as a measure of community distance.

For *de novo* assembly of AM fungal mRNA reads, sequencing reads that were not mapped to the maize genome (i.e., unmapped reads) were collected from all 251 samples and combined into a fastq file, and half of the reads were randomly extracted two times using the SeqKit toolkit (Shen et al., 2016) because the full set of the reads could not be assembled with our standard method. The resultant two read sets were assembled separately with Trinity (ver. 2.4.0) (Grabherr et al., 2011), and then the contigs generated from the two batches were combined and clustered using CD-HIT-EST (Li and Godzik, 2006; Fu et al., 2012) with a 100%-identity cutoff. Average nucleotide length and N50 (the shortest contig length at 50% of the total contig length) were employed as statistics of the assembly. The non-redundant contigs were subjected to Blastx searches at an *E*-value cutoff of 1e^–5^ against the genomes/transcripts of *Rhizophagus irregularis* DAOM181602 (Maeda T. et al., 2018), *R. clarus* HR1 (MAFF520076) (Kikuchi et al., 2014), *Diversispora epigaea* IT104 (Sun et al., 2019), *Gigaspora margarita* BEG34 (Salvioli et al., 2010), *Funneliformis mosseae* DAOM236685, *Acaulospora morrowiae* INVAM-CR315B, *Diversispora versiforme* INVAM-W47540, *Scutellospora calospora* INVAM-IL209, *Racocetra castanea* BEG1, *Paraglomus brasilianum* DAOM240472, *Ambispora leptoticha* INVAM-JA116 (Beaudet et al., 2017), *Saccharomyces cerevisiae* S288c, *Neurospora crassa* OR74A, *Laccaria bicolor* S238N-H82, *Ustilago maydis* 521, *Rhizopus oryzae* 99-892, and *Phycomyces blakesleeanus* NRRL1555 (Supplementary Table 2), as well as against the maize genome and GenBank bacterial genome database, in which contigs that showed the highest similarity to AM fungal sequences were considered as those that originated from AM fungi. From the AM fungal contigs, those containing an open reading frame (ORF) of 50 or longer amino acid residues were predicted using TransDecoder^3^. The high-quality mRNA reads were mapped to the ORF sequences as described in the previous section.

### Statistics

Principal component analysis (PCA) and multiple linear regression analysis were performed using the prcomp and lm functions, respectively, in R (R Core Team, 2021). Scatter plots with 95% confidence intervals were drawn by data analysis with bootstrap estimation in R (Ho et al., 2019). For multiple regression analysis between eigengenes and the soil/plant analytical data (i.e., soil and plant factors), all variables were standardized between −50 (minimum) and +50 (maximum). Multicollinearity was considered in the PCA and multiple regression analysis. In variable selection, the soil and plant factors were first subjected to pairwise correlation analysis, and one of the two factors that were highly correlated (|*r*| ≥ 0.9, Supplementary Table 3) was excluded.

## Results and Discussion

### Characterization of Field Sites/Plots

The sampling plots/sites were characterized by a PCA biplot with the climatic/soil factors (Figure 1C and Supplementary Tables 1, 4). The United States sites are localized in the first quadrant, whereas the Japanese sites are localized in the other quadrants, between which annual precipitation and soil properties were largely different. Among the Japanese sites, the contents of soil organic matter, base, and clay were highly variable. Notably, there were wide gradients of N and P availability among the sites (e.g., NO_3_-N, 10.5–212 mg kg^–1^; Bray II-P, 5.3–494 mg P kg^–1^), which led us to the expectation that there would also be divergent responses of the plants to nutrient deficiency/excess.

### RNA Sequencing, Module Definition, and Preliminary Characterization

By mRNA-Seq, 8.4 ± 0.25 million reads on average were mapped to exons in each sample (Supplementary Table 5). In a preliminary analysis, we focused on the genes involved in mycorrhiza formation because several plant genes that facilitate AM fungal colonization, particularly those involved in arbuscule development/function, are conserved in the plant kingdom (Bravo et al., 2016). We first identified the orthologs of *RAM2* encoding glycerol-3-phosphate acyltransferase, the key enzyme of the mycorrhiza-specific lipid biosynthetic pathway (Bravo et al., 2017), *Pht1;6* encoding the mycorrhiza-inducible Pi transporter (Willmann et al., 2013; Sawers et al., 2017), and *STR2* for primary analysis of expression patterns. The expression levels of *STR2* were strongly correlated with those of *RAM2* (*r* = 0.992) and *Pht1;6* (*r* = 0.939) (Supplementary Figure 1), indicating that the relative expression of these genes was tightly regulated across the genotypes as well as across the ecosystems/countries. This finding led us to conducting WGCNA.

In the WGCNA of the 251 samples, a soft threshold power of 14 provided a scale-free topology of the network (*r*^2^ = 0.91) (Supplementary Figure 2) and resulted in the definition of 19 coexpression modules named with color codes by the WGCNA program (Table 1). These modules were functionally characterized based on enriched genes and Gene Ontology (GO) analysis (Table 1 and Supplementary Table 6): cell division (black), gene expression/translation (blue), cell cycle regulation (green), stress-associated protein quality control (green–yellow), immune response/N assimilation (gray), antioxidation (gray60), branched-chain amino acid (BCAA) metabolism (light cyan), water uptake and diurnal rhythm (light green), immune response (magenta), lipid biosynthesis (midnight blue), root development (pink), two-component and phosphorelay signal transduction systems (purple), response to hypoxia (royal blue), P-starvation response (PSR) (salmon), trehalose biosynthesis (tan), and mycorrhiza formation (yellow). The functions of cyan, dark green, and dark red modules could not be defined by GO analysis or by enriched genes. In the dark green module, only the GO term oxidoreductase activity was overrepresented in addition to enrichment of several genes encoding the enzymes involved in diterpenoid biosynthesis, which was not enough information to characterize this module. No GO terms were enriched in the cyan and dark red modules. In the cyan module, many of the members encoded unknown proteins, so its function could not be defined. In the dark red module, several genes encoding ribosomal proteins were found, but it was also not sufficient information to characterize this module. It is noteworthy, however, that the dark red module showed rather consistent expression levels across the samples (i.e., across genotypes/sites), suggesting that this module has a housekeeping role. Accordingly, the dark red module was employed as control to analyze genotypic differences in module expression.

**TABLE 1:** Enriched Gene Ontology (GO) terms and putative function of gene coexpression modules.

Module (no. of gene)	Enriched GO term^†^	Putative function
Black (1,574)	DNA packaging, cellular component biogenesis, translation, RNA modification, auxin transport	Cell division
Blue (4,134)	RNA splicing, ribonucleoside catabolic process, regulation of translation	Gene expression/translation
Cyan (86)	(No enrichment)	(Undefined)
Dark green (46)	Oxidoreductase activity	(Undefined)
Dark red (51)	(No enrichment)	(Undefined)
Green (1,257)	Mitotic cell cycle, protein localization, organelle organization	Cell cycle regulation
Green-yellow (139)	Protein folding, response to stress, regulation of protein stability	Stress-associated protein quality control
Gray (8,460)	Defense response, cellular amino acid metabolic process, immune response, response to nitrate	Immune response and N assimilation
Gray60 (77)	Antioxidant activity, oxidoreductase activity	Antioxidation
Light cyan (81)	Branched-chain amino acid catabolic process, mitochondrial matrix	BCAA metabolism
Light green (67)	Water transport, rhythmic process	Water uptake and diurnal rhythm
Magenta (397)	Defense response, immune response, response to other organisms	Immune response
Midnight blue (82)	Lipid metabolic process, fatty acid metabolic process	Lipid biosynthesis
Pink (471)	Cell wall biogenesis, lignin metabolic process, root morphogenesis	Root development
Purple (275)	Phosphorelay response regulator activity	Two-component and phosphorelay signal transduction systems
Royal blue (60)	Response to decreased oxygen levels, lactate biosynthetic process	Response to hypoxia
Salmon (101)	Cellular response to phosphate starvation, phosphate ion transport, acid phosphatase activity, galactolipid biosynthetic process	P-starvation response
Tan (131)	Trehalose biosynthetic process	Trehalose biosynthesis
Yellow (1,023)	Lipid biosynthetic process, terpenoid biosynthetic process, chitinase activity	Mycorrhiza formation

*^†^All GO terms enriched in the modules are listed in Supplementary Table 6.*

In the subsequent analysis, we further characterized the mycorrhizal, PSR, and root development modules, because they are likely to be directly involved in nutrient foraging. We had also realized, however, that a module responsive to N starvation was not clearly defined in this analysis, which is addressed in a later section.

### Mycorrhizal Module

The majority of genes known to participate in arbuscule development and functioning are enriched in this module; e.g., genes encoding the transcription factors RAM1 (Gobbato et al., 2012), RAD1 (Park et al., 2015), and WRI5 (Jiang et al., 2018), Pi transporter Pht1;6, nitrate transporter NPF4.5 (Wang et al., 2020), ammonium transporter AMT3;1 (Koegel et al., 2013), H^+^-ATPase HA1 (Krajinski et al., 2014; Wang et al., 2014), key enzymes for mycorrhiza-specific lipid biosynthesis, RAM2 and FatM (Bravo et al., 2017), putative lipid exporters STR and STR2, and enzymes involved in strigolactone/mycorradicin/blumenol biosynthesis, DXS2 (Walter et al., 2002; Floss et al., 2008), PSY2 (Stauder et al., 2018), CCD7, CCD8, D27, and CCD1 (Al-Babili and Bouwmeester, 2015) (Supplementary Table 7).

In the WGCNA, the expression levels of the modules are represented by eigengenes (PC1 scores) (Supplementary Table 8), and genes that show a higher correlation coefficient between the eigengenes and their expression levels (i.e., those with higher connectivity) are, in general, considered as those that play a more important role in the module (Langfelder and Horvath, 2008). In this module, *STR2* (Zm00001d043722) showed the highest connectivity (*r* = 0.977) (Supplementary Table 7). To assess the regulatory robustness of the module across all genotype-site combinations, the module member genes were sorted by connectivity, and the expression levels of the 1st (Zm00001d033915), 50th (Zm00001d033002), and 100th (Zm00001d032267) percentile genes relative to those of *STR2* were plotted (Supplementary Figure 3). The levels of the 1st percentile gene were constant across the genotype-site combinations, whereas those of the 50th and 100th percentile genes showed variability in several combinations, suggesting that the relative expression levels of lower-connectivity genes may be finely and differently modulated at least in some genotypes. To address genotypic differences, the absolute expression levels (i.e., eigengenes) of the two genotypes, P2023 and LG2533, grown in the Sapporo site (Supplementary Tables 4, 8) and those of another pair of genotypes, Canberra 90EX and P2088, grown in the Nagoya site were compared with reference to the dark red module (Supplementary Figure 4). Even in the same sites, the absolute expression levels of the mycorrhizal module were different between the genotypes to some extent, which could be due to the difference in nutrient status (Supplementary Table 4) and/or differentiation in mycorrhizal dependency among the genotypes (e.g., Sawers et al., 2017).

To characterize this module in relation to AM fungal colonization/functionality, we analyzed the “unmapped sequence reads” (i.e., those that were not mapped to the maize genome) that might contain AM fungal RNA reads. Generally, sequence reads obtained by mRNA-Seq contain a small number of those that originated from rRNA, which led us to the idea that the abundance of AM fungal rRNA reads relative to plant rRNA read number could represent relative fungal biomass in the roots (Supplementary Table 9). It was considered, however, that fungal rRNAs that have A-rich regions were preferentially sequenced in the mRNA-Seq, because polyA-tailed RNA was purified prior to library construction, which might bias fungal rRNA read abundance and composition across the samples. To evaluate the severity of these biases in the mRNA-Seq, we chose randomly 20 samples from the 251 samples and conducted rRNA-Seq, which would provide unbiased rRNA read counts (Supplementary Table 10). The correlation analysis indicated that the rRNA read counts obtained by the mRNA-Seq were highly correlated with those obtained by the rRNA-Seq, with a correlation coefficient of 0.919 (*P* < 0.001) (Supplementary Figure 5), indicating that the bias in read abundance is minimum. On the other hand, NMDS showed that OTU compositions in several samples were largely different between those obtained by the two sequencing methods (Supplementary Figure 6), indicating that the bias in read composition is significant in some cases. Accordingly, we employed only the abundance data, but not the compositional data, for subsequent analyses. The unmapped reads were also *de novo* assembled, and 427,813 out of 2,495,764 non-redundant contigs were predicted as those that originated from AM fungal genes: average length, 461 bp; N50, 525 bp (Supplementary Figure 7). We searched the contigs that showed similarity to the putative Pi exporter *SYG1-1*, polyphosphate polymerase *VTC4*, vacuolar Pi exporter *PHO91*, and aquaglyceroporin *AQP3*, which are likely to be involved in Pi delivery in the fungi (Ezawa and Saito, 2018). Then we identified 86, 74, 159, and 105 contigs similar to *SYG1-1*, *VTC4*, *PHO91*, and *AQP3*, respectively, mapped the unmapped reads to these contigs, combined read counts within each gene, and normalized on the basis of TPM of the plant (Supplementary Table 11). These expression data were subjected to multiple linear regression analysis together with the AM fungal rRNA read counts using the mycorrhizal module eigengenes as an objective variable. Among them, the read counts of rRNA, *SYG1-1*, and *PHO91* were significant explanatory variables for the eigengenes with correlation coefficients of 0.875, 0.15, and 0.233, respectively (*R*^2^ = 0.782, *P* < 0.001) (Supplementary Figure 8), suggesting that the module eigengenes reflect functional colonization of the fungi.

**FIGURE 2:**
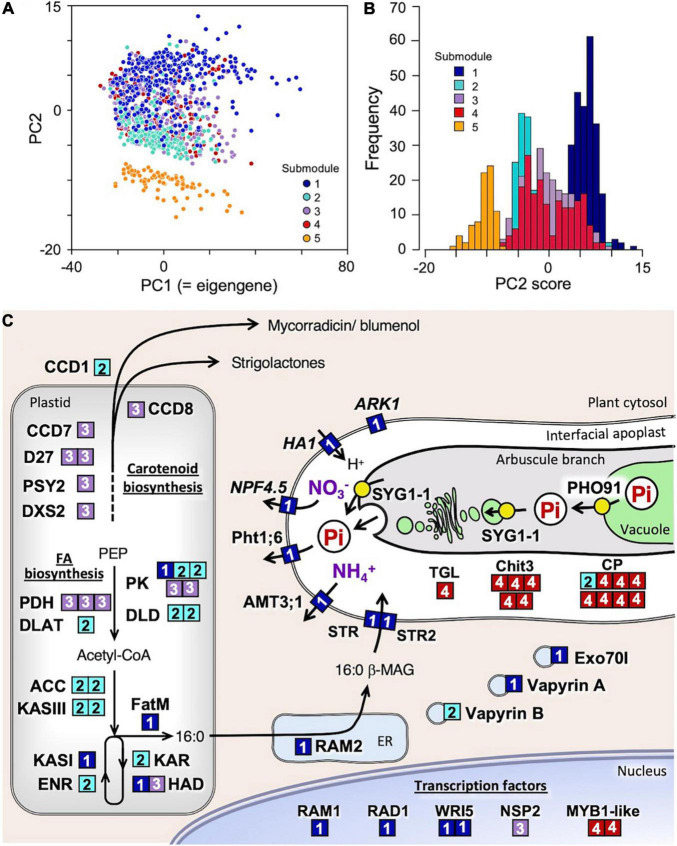
Functional categorization of the 1,023 genes assigned to the mycorrhizal module and into five submodules by k-means clustering analysis using correlation-based distance as a measure. **(A)** Principal component analysis (PCA) plot of genes in submodules 1–5 and **(B)** frequency distribution of PC2 score of the genes. **(C)** Putative function and cellular localization of genes whose orthologs have been functionally characterized in previous studies. The number of the submodule to which the genes belong is indicated in the boxes with same colors in panels **(A,B)**. No orthologs of the genes in submodule 5 have so far been functionally characterized; thus, they are excluded from this scheme.

For further functional categorization of the 1,023 genes of this module, k-means clustering analysis was performed using correlation distance as a measure. Clustering the genes into five submodules successfully separated them into different functional groups along the principal component 2 (PC2) axis of the PCA performed using the expression data of all the module member genes (Figure 2A, Table 2, and Supplementary Table 12). In submodule 1, the majority of the essential components of arbuscule development and nutrient exchange were enriched, e.g., *RAM1*, *RAD1*, *WRI5*, *Pht1;6*, *NPF4.5*, *AMT3;1*, *HA1*, *RAM2*, *FatM*, *STR*/*STR2*, and serine/threonine receptor-like kinase ARK1 (Roth et al., 2018), and genes involved in membrane trafficking *EXO70I* (Zhang et al., 2015) and *Vapyrin A* (Pumplin et al., 2010) (Figure 2C and Supplementary Table 7). The enrichment of these genes suggests that submodule 1 plays a central role in nutrient exchange across the periarbuscular membrane. In submodule 2, the GO terms fatty acid biosynthetic process and plastid were overrepresented, reflected in the enrichment of genes encoding enzymes involved in *de novo* biosynthesis of C16:0-fatty acid *via* acetyl-CoA in plastids. This suggests that submodule 2 has a role in the biosynthesis of an essential component of lipids for the construction of the periarbuscular membrane and/or for export to the fungi. In submodule 3, the GO terms isoprenoid biosynthetic process, carotenoid metabolic process, and hormone metabolic process were overrepresented; genes involved in the biosynthesis of carotenoid derivatives *DXS2*, *PSY2*, *CCD7*, *CCD8*, and *D27* were enriched. In addition, the GRAS transcription factor NSP2 that regulates strigolactone biosynthesis by modulating *D27* expression (Liu et al., 2011) also belongs to this submodule. Therefore, it is likely that submodule 3 regulates the early processes of fungal accommodation by strigolactone production. In submodule, 4 the GO terms senescence-associated vacuole, amino sugar metabolic process, and extracellular space were overrepresented. The enrichment of genes encoding an MYB1-like transcription factor, chitinase, and cysteine protease suggests that this submodule is responsible for arbuscule degeneration/turnover (Floss et al., 2017). Neither GO terms nor known genes involved in fungal accommodation/functioning were enriched in submodule 5; therefore, the biological function of this submodule could not be defined. The average connectivity of submodules 1–5 with mycorrhizal module eigengenes was 0.88, 0.72, 0.77, 0.75, and −0.59, respectively. In addition, most genes in the submodule 1 showed positive PC2 scores, while most of those in submodules 2 and 5 showed negative PC2 scores (Figure 2B). These results suggest that the expression of each submodule is finely and adaptively tuned in response to the environment.

**TABLE 2:** Enriched Gene Ontology (GO) terms and putative function of the submodules of the mycorrhizal module.

Module (no. of gene)	Enriched GO term^†^	Putative function
Submodule 1 (332)	Peptidase activity, transferase activity	Nutrient exchange
Submodule 2 (203)	Fatty acid biosynthetic process, plastid part	Fatty acid biosynthesis
Submodule 3 (219)	Terpenoid biosynthetic process, carotenoid metabolic process, plastid part	Carotenoid biosynthesis
Submodule 4 (189)	Amino sugar metabolic process, defense response to fungus, extracellular space	Arbuscule degeneration
Submodule 5 (80)	(No enrichment)	(Undefined)

*^†^All GO terms enriched in the modules are listed in Supplementary Table 12.*

### Phosphate-Starvation Response Module

This module consists of 101 genes, in which the GO terms involved in PSR, e.g., cellular response to Pi starvation, Pi transport, acid phosphatase activity, and galactolipid biosynthetic process, were overrepresented (Supplementary Table 6). Pht1;3, one of the four Pi transporters in this module, is likely to be responsible for the root-direct pathway (Glassop et al., 2005; Nagy et al., 2006) in which the transporter is localized to the root epidermis and mediates Pi uptake independently of the mycorrhizal pathway (Smith et al., 2011). Six genes encoding SPX (SYG1-PHO1-XPR1) domain, a sensor for the inositol polyphosphate-mediated signaling pathway for the maintenance of Pi homeostasis in eukaryotes (Wild et al., 2016), were also enriched in this module; one encodes the Pi exporter PHO1 (Wege et al., 2016) and the other five encode SPX-domain containing proteins. Many acid phosphatase genes, including those encoding purple acid phosphatase, were assigned to this module. Two orthologs of *NIGT1* that encode an MYB-type transcription factor belong to this module. NIGT1 was originally identified as a repressor of NO_3_^–^ uptake (Maeda Y. et al., 2018) but was found to be playing a role in PSR by modulating the expression of SPX genes (Medici et al., 2019; Ueda et al., 2019; Hu and Chu, 2020). It has been well-documented that the transcription factor PHOSPHATE STARVATION RESPONSE (PHR) plays a central role in PSR as a master regulator in Arabidopsis, a non-mycorrhizal plant (Rubio et al., 2001), but recently, one of the orthologs, PHR2, was found to be also involved in the regulation of mycorrhiza formation in rice (Shi et al., 2021; Das et al., 2022) and *Lotus japonicus* (Das et al., 2022). In maize, so far, two orthologs of PHR have been found (Calderón-Vázquez et al., 2011), but both were assigned to the gene expression/translation module in this study (Supplementary Table 7), indicating the complexity of the PHR-mediated regulatory mechanism. In higher plants, including maize, Pi deficiency induces the replacement of membrane phospholipids with non-phosphorous lipids such as galactolipids and sulfolipids (Tjellström et al., 2008). Genes encoding glycerophosphodiester phosphodiesterase (GDPD2) for degradation of phospholipid, monogalactosyldiacylglycerol synthase 2 (MGDG2) for biosynthesis of galactolipids, and sulfoquinovosyl transferase (SQD2) for biosynthesis of sulfolipids were assigned to this module.

*MGDG2* (Zm00001d031428) showed the highest connectivity, and that of *SQD2*, *GDPD2*, *NIGT1*, and genes encoding SPX-domain containing proteins and purple acid phosphatases was also comparable to the connectivity of *MGDG2* (Supplementary Table 7). The expression levels of the 5th (Zm00001d026156), 50th (Zm00001d043681), and 100th (Zm00001d020985) percentile genes relative to *MGDG2* expression were generally constant even in the lowest connectivity gene (Supplementary Figure 9), indicating that the relative expression levels of the genes are tightly regulated across the genotypes/sites. Genotypic differences in absolute expression level also seemed minimum in this module (Supplementary Figure 4).

### Root Development Module

This module consists of 471 genes, and GO terms involved in cell wall synthesis and root development were overrepresented; e.g., plant cell wall biogenesis, cellulose synthase, cytoskeleton, root system development, root morphogenesis, and root epidermal cell differentiation (Supplementary Table 6). This indicates that this module is responsible for the development of the root system, accompanying transcriptional activation of a set of genes for cell wall biogenesis. The plant cell wall is composed of the primary cell wall that supports the fundamental growth of cells and the secondary cell wall that supports the primary cell wall mechanically and facilitates water transport. CesA genes encode a cellulose synthase catalytic subunit and form cellulose synthase complexes that consist of 18–24 *CesA* proteins; CesA1, CesA2, CesA3, CesA5, CesA6, and CesA9 are involved in primary cell wall synthesis, while CesA4, CesA7, and CesA8 are for secondary cell wall synthesis in Arabidopsis (Taylor et al., 2003; Desprez et al., 2007; Persson et al., 2007). In the maize genome, 20 *CesA*s have been found (Penning et al., 2009), among which 11 genes, *CesA1*, *CesA2*, *CesA4*, *CesA6*, *CesA7a*, *CesA7b*, *CesA9*, *CesA10*, *CesA11*, *CesA12a*, and *CesA12b* are assigned to this module (Supplementary Table 7). In addition to the *CesA*s, many genes encoding polysaccharide-biosynthetic/modification enzymes, e.g., glycosyltransferases and glucanases, were also found in this module. There are 15 genes encoding the essential parts of the cytoskeleton, alpha- and beta-tubulins, and a microtubule motor protein in this module. Eighteen transcription factors, e.g., an ethylene-responsive transcription factor (ERF), five NAC-domain transcription factors, and seven MYB-domain transcription factors, are found in this module. ERF035 was expressed in regions related to cell division/differentiation, e.g., lateral roots and central cylinder of primary roots, and upregulate *CesA1* in Arabidopsis (Saelim et al., 2019). It has been known that many NAC- and MYB-domain transcription factors are responsible for the regulation of secondary cell wall synthesis (Yamaguchi et al., 2008; Nakano et al., 2015) and cell division (Willemsen et al., 2008) during root formation. It is noteworthy that the expression of several genes involved in root hair initiation/elongation, *ROOT HAIRLESS 1* (*RTH1*) (Wen et al., 2005), *RESPIRATORY BURST OXIDASE HOMOLOG 1* (*RBOH1*) (Mangano et al., 2017), and two *ROOT HAIR DEFECTIVE 3* (*RDH1*) (Schiefelbein and Somerville, 1990) that belong to other modules, was also positively correlated with the expression of this module (*P* < 0.05) and, interestingly, negatively correlated with that of the mycorrhizal module (*P* < 0.001) (Supplementary Table 7). In addition, the expression levels of 40 genes involved in auxin response and transport (e.g., those encoding auxin responsive protein/factor and auxin transporter/carrier), although most of them belong to the modules of immune response and N assimilation and cell division, were positively correlated with the eigengenes of the root development module (*P* < 0.05).

A gene encoding endoglucanase 2 (*EG2*, Zm00001d021304) showed the highest connectivity in the module (Supplementary Table 7). The expression levels of the 5th (Zm00001d010976), 50th (Zm00001d042276), and 100th (Zm00001d006756) percentile genes relative to *EG2* expression were quite constant across the genotypes/sites (Supplementary Figure 10), indicating strict regulation of the module member genes. Genotypic differences in absolute expression levels seemed likely to be minimum, although the expression levels were rather variable among the individuals of the same genotypes (Supplementary Figure 4).

### Nitrogen Starvation Response Module

To explore the N starvation response module, we first searched for genes involved in the initial steps of N assimilation, and six *AMT*s, three *NRT1*s, four *NRT2*s, five genes encoding NRT1/PRT family protein (NPF), five nitrate reductase (NR) genes, one nitrite reductase (NIR) gene, six glutamine synthetase (GS) genes, four glutamate synthase (GOGAT) genes, and one glutamate dehydrogenase (GDH) gene were found to be expressed in the roots (Supplementary Table 13). Among the 36 genes, six from the immune response/N assimilation (gray) module and four from the mycorrhizal module showed significant negative correlations with soil NO_3_-N levels (*P* < 0.05), implying that the 10 genes were upregulated in response to low NO_3_^–^ levels. The PC1 scores of the ten NO_3_^–^-responsive genes were calculated based on their expression levels, and genes whose expression levels were correlated with the PC1 scores were extracted from all genes at a criterion of | *r*| > 0.5 (*P* < 1e^–17^) and tentatively grouped as a low-NO_3_^–^ response module (Supplementary Table 7). This module consisted of 1,436 genes, of which 982 were of the mycorrhizal module. The remaining 454 genes were those whose expression levels were highly correlated with the eigengenes of the mycorrhizal module, including two genes encoding GS root isozyme and the transcription factor ROOTLESS WITH UNDETECTABLE MERISTEMS 1 (RUM1) that initiates lateral root formation (Woll et al., 2005). The eigengenes of the low-NO_3_^–^ response module, therefore, showed a strong positive correlation with those of the mycorrhizal module (*r* = 0.997). The pairwise correlation analysis between the eigengenes of the low-NO_3_^–^ response module and soil/plant factors (Supplementary Table 14), as well as the PCA biplot constructed with the correlation coefficients of the modules for the soil/plant factors (Supplementary Figure 11), indicated that the response patterns of the low-NO_3_^–^ response module to the factors were quite similar to those of the mycorrhizal module. The analyses strongly suggested that the genetic module that plays a main role in N uptake under low-NO_3_^–^ conditions is the mycorrhizal module; thus, the low-NO_3_^–^ response module was not considered in subsequent analyses.

In contrast to NO_3_^–^, soil NH_4_-N levels were generally low across the plots/sites (<10 mg-N kg^–1^), except for those in the Kasai site (Supplementary Table 4). Accordingly, the range of soil NH_4_-N level was too narrow to explore; thus, a module responsive to low-soil NH_4_^+^ was not considered.

### Drivers of the Foraging Modules and Module–Module Interplays

**FIGURE 3:**
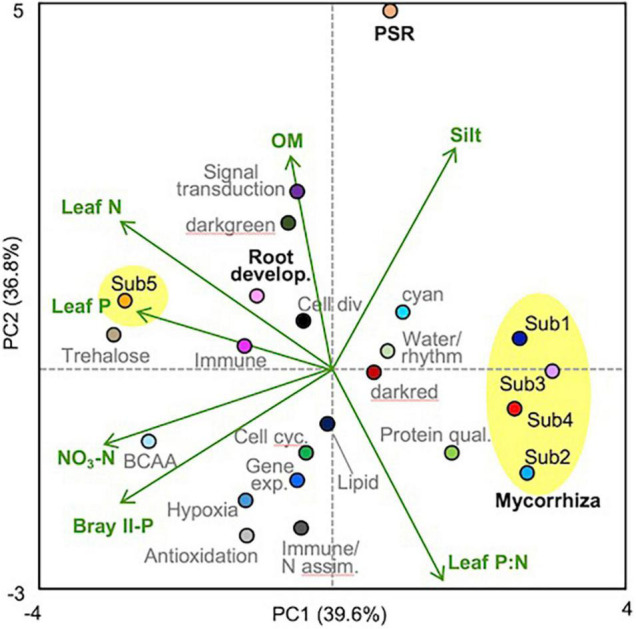
PCA biplot of module-factor correlations. The plot was drawn based on the correlation coefficients obtained by pairwise correlation analysis between the soil/plant factors and the module eigengenes (Supplementary Table 14), in which the factors leaf N, P, and P:N, Bray II-P, NO_3_-N, organic matter (OM), and silt% were selected by taking into account multicollinearity. The submodules of the mycorrhizal module were circled with yellow. Module names (functions) and colors are listed in Table 1, and the mycorrhizal (submodules), PSR, and root development modules were indicated with bold black letters.

Pairwise correlation coefficients between module eigengenes and soil/plant factors were calculated (Supplementary Table 14), and a module-factor PCA biplot was constructed based on the coefficients, considering multicollinearity among the factors (Figure 3). Submodules 1–4 of the mycorrhizal module showed positive scores along the PC1 axis that explained 39.6% of the variation and were associated with lower Bray II-P and NO_3_-N in the soil and with lower leaf P and N concentrations, whereas submodule 5 and the root development module showed negative PC1 scores. The PSR module showed a high positive score along the PC2 axis that explained 36.8% of the variation and was associated with higher contents of organic matter and silt and lower leaf P:N ratios. Submodules 1 and 5 and the root development module also showed positive PC2 scores, while submodules 2 and 4 showed negative PC2 scores. To support the PCA biplot, a multiple linear regression analysis of the module eigengenes was also conducted using all factors as explanatory variables, except for one of the two factors that showed a correlation coefficient (|*r*|) of more than 0.9 (Supplementary Table 3). Bray II-P, NO_3_-N, and clay% were the major negative factors for the mycorrhizal module among the soil factors (Table 3). Bray II-P and exchangeable Ca were negative factors for the PSR module, while organic matter was a major positive factor for the module. For the root development module, clay% was a positive factor, and organic matter and exchangeable Mg were negative factors. As expected from the PCA, leaf P was a strong negative factor for the mycorrhizal module, a positive factor for the root development module, and was not a significant factor for the PSR module. Leaf P:N ratios showed contrasting effects on the mycorrhizal and PSR modules; the ratio was a strong positive driver for the mycorrhizal module but a negative driver for the PSR module. The expression levels of the mycorrhizal and PSR modules were higher in plants with larger stem diameters and in those with slower growth rates, whereas the root development module was downregulated in plants with larger stem diameters.

**TABLE 3:** Coefficients of the soil and plant factors with the eigengenes of mycorrhizal, phosphate starvation response (PSR), and root development modules in multiple regression analysis.

		Module	
Factor	Mycorrhiza	PSR	Root development
**Soil factor**			
pH	0.072	0.200**	0.078
OM	0.149*	0.476***	−0.199**
Bray II-P	−0.198**	−0.276***	−0.068
NO_3_-N	−0.294***	0.021	−0.056
K	0.040	0.276***	−0.025
Mg	−0.102	−0.069	−0.237***
Ca	−0.118	−0.533***	0.130
Silt%	−0.165*	0.182**	0.092
Clay%	−0.487***	0.058	0.394***
**Plant factor**			
Stem diameter	0.469***	0.329**	−0.274**
Growth rate	−0.400**	−0.676***	0.126
Leaf P	−0.854***	−0.153	0.369**
Leaf N	0.019	0.019	0.207
Leaf P:N	0.641***	−0.401**	−0.258
Intercept	−4.158	−42.407***	−16.572**
*r* ^2^	0.642***	0.670***	0.441***

Asterisks indicate significant levels (Student’s t-test): *P < 0.05; **P < 0.01; ***P < 0.001.

**FIGURE 4:**
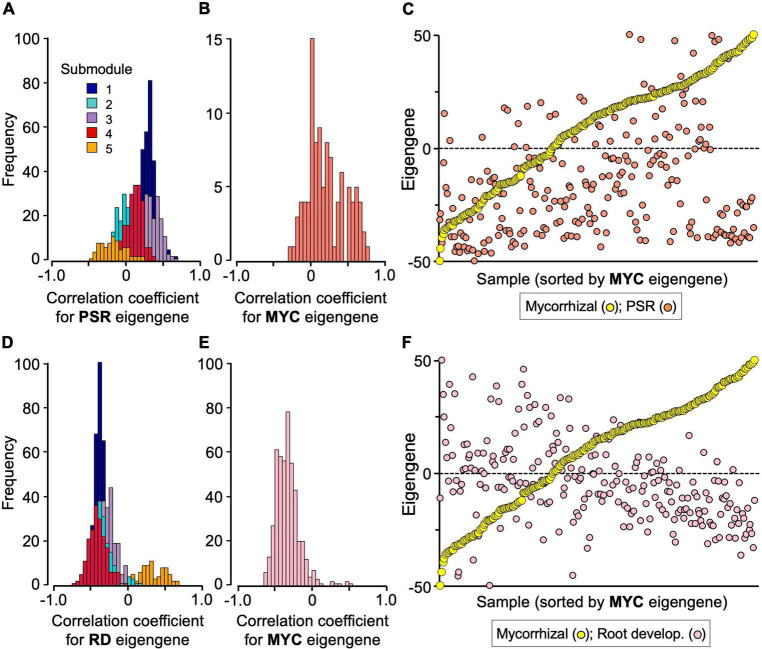
Interplay of the mycorrhizal (MYC) module with the PSR and root development (RD) modules. **(A)** Frequency distributions of correlation coefficients of the mycorrhizal submodule genes with PSR module eigengenes, and **(B)** those of the PSR module genes with mycorrhizal module eigengenes. **(C)** Scatter plot of the eigengenes of the mycorrhizal and PSR modules of the 251 samples, in which the samples were sorted by the order of mycorrhizal module eigengenes. **(D)** Frequency distributions of correlation coefficients of the mycorrhizal submodule genes with the root development module eigengenes, and **(E)** those of the root development module genes with the mycorrhizal module eigengenes. **(F)** Scatter plot of the eigengenes of the mycorrhizal and root development modules of the 251 samples, in which the samples were sorted by the order of mycorrhizal module eigengenes. The data were extracted from Supplementary Tables 7, 8, and all the eigengenes were standardized between –50 (minimum value) and +50 (maximum value) for plotting. The submodule numbers of the mycorrhizal module genes are indicated with the following colors: 1, dark blue; 2, turquoise; 3, purple; 4, red; 5, orange.

To analyze module–module interplays, a gene–eigengene correlation analysis was conducted. Among the 1,023 genes of the mycorrhizal module, 598 genes, most of which belong to submodules 1 and 3, showed positive correlation coefficients with the PSR-module eigengenes (*P* < 0.01) (Figure 4A and Supplementary Table 7). Similarly, 51 out of the 101 PSR module genes, including most of the acid phosphatase and Pi transporter (Pht1) genes, also showed positive correlation coefficients with the mycorrhizal module eigengenes (*P* < 0.01) (Figure 4B and Supplementary Table 7). However, these correlations were ambiguous in the simple sample-eigengene plot of the two modules probably because of their partial correlations (Figure 4C). Clear negative correlations were observed between the mycorrhizal module and the root development module; the expression levels of 873 genes of the mycorrhizal module were negatively correlated with the eigengenes of the root development module, and 414 out of the 471 genes of the root development module showed negative correlation coefficients with the mycorrhizal module eigengenes (*P* < 0.01) (Figures 4D,E and Supplementary Table 7). In fact, the correlation coefficient between the eigengenes of the two modules was −0.439 (*P* < 0.001), as reflected in the simple sample-eigengene plot (Figure 4F). No significant correlations were observed between the PSR and root development modules (Supplementary Figures 12A–C and Supplementary Table 7).

As proposed by the first hypothesis, three genetic modules, mycorrhiza formation, PSR, and root development that were likely to be directly involved in foraging strategies, were identified. The mycorrhizal module was upregulated by P and N deficiencies in the plants, as well as by low availabilities of P and N in the soil. In contrast, the root development module responded in the opposite direction. The PSR module was mainly driven by P deficiency relative to N (i.e., leaf P:N ratios), supporting the second hypothesis. However, no specialized genetic module for N starvation response could be identified. Although a set of low-NO_3_^–^ responsive genes was identified, most of the genes belonged to the mycorrhizal module in addition to those co-expressed with the mycorrhizal module. These results strongly suggest that, at least under NO_3_^–^ depleted conditions, maize largely relies on mycorrhizae for NO_3_^–^ uptake, as proposed in rice (Wang et al., 2020).

We consider that the differential role of mycorrhiza in the uptake of organic P and N differentiates the plant responses to P and N deficiencies. Organic P cannot be taken up directly either by plants or by AM fungi (Shen et al., 2011). Accordingly, plants (Plaxton and Tran, 2011) and AM fungi (Joner et al., 2000; Koide and Kabir, 2000; Sato et al., 2019) evolved genes encoding phosphatases for direct mineralization and, for indirect mineralization, associated with P-solubilizing bacteria in the rhizosphere and the hyphosphere (e.g., Richardson and Simpson, 2011; Zhang et al., 2016). In contrast, although both plants and AM fungi are capable of uptaking organic N such as amino acids, the contribution of the root-direct pathway to amino acid uptake seems to be much smaller than that of the mycorrhizal pathway. This assumption is supported by the following two observations. First, amino acids in the rhizosphere turn over so rapidly that they never reach the root surface, whereas extraradical mycelia of the fungi could access amino acids in the bulk soil beyond the rhizosphere (Jones, 1999). Second, amino acid uptake by roots would be primarily for retrieval of amino acids that leaked out of root cells because the efflux of amino acids from roots is not negligible and frequently exceeds their influx (Nashölm et al., 1998; Näsholm et al., 2009). Investment in mycorrhizae, therefore, is likely to be a more efficient strategy for N acquisition under inorganic N-limited conditions.

Water availability greatly affects the efficiency of nutrient uptake. The Pi taken up by AM fungal hyphae is translocated toward the roots by water flow through the hyphae, which is primarily driven by host transpiration (Kikuchi et al., 2016). NO_3_^–^ is highly mobile in soil, but the mass flow driven by transpiration sustains NO_3_^–^ uptake by roots (McMurtrie and Näsholm, 2018). In this context, it was expected that genes encoding water channels would be enriched in nutrient foraging modules to regulate water uptake. Plasma membrane aquaporins (plasma membrane intrinsic protein, PIP) are mainly responsible for water transport across the plasma membrane (Kapilan et al., 2018), but six out of 13 PIP genes were enriched in the water uptake/diurnal rhythm module that is regulated independently from the nutrient foraging modules (Figure 3). Furthermore, most of the PIP genes were downregulated by mycorrhiza formation (Supplementary Table 7). Instead, four out of seven genes encoding nodulin 26-like membrane intrinsic protein (NIP) were enriched in the mycorrhizal module. NIP was originally identified as a major component of the peribacteroid membrane in soybean root nodules (Rivers et al., 1997) and takes up ammonia from the symbiotic interface (Niemietz and Tyerman, 2000). The NIP family is a group of aquaglyceroporin unique to plants (Wallace et al., 2006), and in normal roots, a NIP is localized in the plasma membrane and transports a variety of uncharged solutes, e.g., arsenite (Ma et al., 2008), silicon (Ma et al., 2006), boron (Takano et al., 2006), and urea (Yang et al., 2015), with low or no water permeability (Wallace et al., 2006). Alteration of the expression pattern of aquaporin genes by mycorrhiza formation has widely been studied in gramineous plants in the context of drought tolerance (e.g., Bárzana et al., 2014; Quiroga et al., 2019; Symanczik et al., 2020), but the upregulation of NIPs seems to be involved in ammonia/ammonium uptake from the arbuscular interface (Uehlein et al., 2007). It is likely that the water uptake capability for nutrient acquisition would be tuned at the posttranslational level rather than at the transcriptional level (Chaumont and Tyerman, 2014).

**FIGURE 5:**
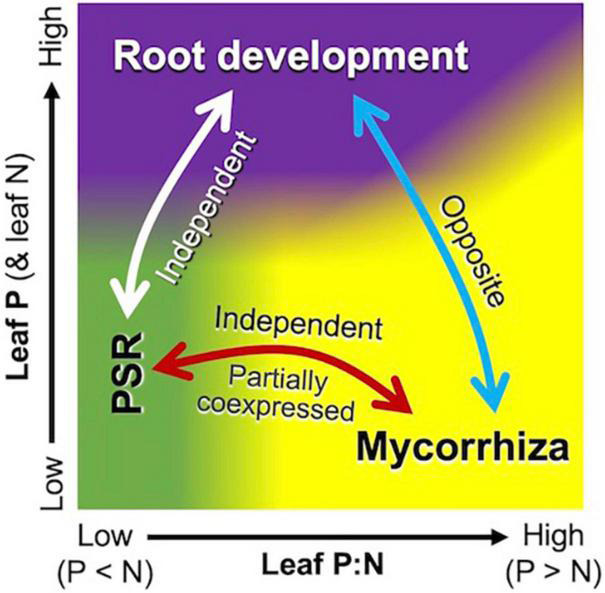
Schematic representation of interplay among the mycorrhizal (yellow area), PSR (green area), and root development (purple area) modules with respect to plant nutrient status. Leaf P:N ratios mainly drive the mycorrhizal module positively and the PSR module negatively, although parts of the genes in the two modules are coexpressed. Higher leaf P (and N) concentrations upregulate the root development module and downregulate the mycorrhizal module, but N deficiency under P-sufficient conditions leads to higher P:N ratios and thus upregulates the mycorrhizal module.

Phosphate-starvation response and mycorrhiza formation are two major strategies for the acquisition of P in plants, but their interplay has attracted interest only recently (Shi et al., 2021; Yaffar et al., 2021; Das et al., 2022; Han et al., 2022). The regulatory role of PHR2, both in PSR and mycorrhiza formation (Shi et al., 2021; Das et al., 2022) was partially supported by the coexpression of some PSR genes with mycorrhizal submodules 1 and 3. It is noteworthy, however, that leaf P:N ratios drive the PSR module negatively and the mycorrhizal module positively (Figure 5), suggesting that these two modules are regulated not solely by PHR2 but also at multiple levels. This differential response of the PSR module could be interpreted by the high connectivity to *NIGT1*. Although this transcription factor is involved in the signaling cascade of PSR (Maeda Y. et al., 2018; Ueda et al., 2019), it is upregulated in a NO_3_^–^-concentration-dependent manner (Maeda Y. et al., 2018). This implies that increases in soil NO_3_^–^ increase the expression of the PSR module by decreasing leaf P:N ratios, under which the mycorrhizal module is downregulated. The independence of the PSR module from the mycorrhizal module would facilitate an alternative (backup) strategy to cope with P deficiency when P delivery from the mycorrhizal pathway does not meet the demand because of, e.g., low population density of AM fungi, extremely low Pi availability, and presence of excess N in soil (Shi et al., 2021; Das et al., 2022).

The present study demonstrated that resource allocation between roots and mycorrhizae is coordinately, rather than independently, regulated according to above-ground nutrient levels at the transcription level. The negative correlation in the expression of the root development module and the mycorrhizal module (as a function of leaf nutrient level) suggests that root development is intrinsically an opposite strategy of mycorrhizae for foraging (Figure 5). It has been well-documented that increases in soil nutrient availability decrease the percent root length colonized by AM fungi by improving plant nutrient status (e.g., Menge et al., 1978), representing decreases in AM fungi-to-root biomass ratios. This modulation of relative fungal biomass in response to nutrient availability has traditionally been interpreted as dependency on fungi (Treseder, 2013) but rarely in the context of root-mycorrhiza interplay as foraging strategies. Recently, for categorizing root resource acquisition strategies, a framework of root economic space, which is defined by mycorrhizal dependency (“collaboration gradient,” 1st dimension) and slow/fast resource return on investment (“conservation gradient,” 2nd dimension), has been proposed (Bergmann et al., 2020). Interestingly, maize is located in the middle of the collaboration gradient, suggesting that maize has balanced strategies for resource acquisition *via* the root-direct and mycorrhizal pathways. The clear shifts between the root development and mycorrhizal modules along the leaf/soil nutrient gradients are likely to reflect the balanced strategies.

The impact of mycorrhiza formation on root architecture has extensively been studied, demonstrating that the interactions are quite complex and regulated at multiple levels (Gutjahr and Paszkowski, 2013). Mycorrhiza formation promotes localized proliferation of lateral roots (Fusconi, 2013; Gutjahr and Paszkowski, 2013; Gutjahr et al., 2015; Yu et al., 2016; Chen et al., 2017), which is triggered by pre-symbiotic signals released from germinating spores and in response to local increases in nutrients around arbuscules (Gutjahr and Paszkowski, 2013). In this study, we observed that *RUM1*, a key regulator of lateral root formation, was coexpressed with the mycorrhizal module, supporting previous observations. Furthermore, *RTH1*, *RBOH1*, and two *RDH1* that regulate root hair formation were found to be downregulated with increasing expression of the mycorrhizal module, adding further complexity to the root morphological/architectural responses to mycorrhiza formation. Modification of root hair development, however, was not examined in this study and needs to be confirmed experimentally.

In the Early Devonian, AM symbiosis facilitated the terrestrialization of early plants that only had a poor root system by providing a water/nutrient uptake pathway (Humphreys et al., 2010). During the Middle to Late Devonian, plants evolved a substantial root system not only for taking up water/nutrients but also for anchoring the body to the soil (Kenrick and Crane, 1997). We consider that this dual functionality of the roots drove the development of the fine-tuning system for the mycorrhizal and root-direct pathways. In terms of water/nutrient uptake, these two pathways are functionally redundant. Their roles, however, could be interpreted by the cost-benefit trade-off between the enlargement of surface area for nutrient uptake and the rates (efficiency) of nutrient uptake/translocation (Smith et al., 2011). The mycorrhizal pathway is mediated by fungal hyphae that are much finer than roots and longer than root hairs, which provide a larger surface area per unit carbon investment and thus enable exploration of a larger soil volume beyond the P depletion zone (e.g., Rhodes and Gerdemann, 1975). Therefore, under nutrient-depleted conditions where diffusion rates of nutrients toward roots are slow, plants invest more in the mycorrhizal pathway, because hyphal foraging provides more rapid nutrient capture/translocation than the root-direct pathway. In contrast, the root-direct pathway may facilitate more rapid uptake and translocation of nutrients under nutrient-enriched conditions in which the diffusion rates of nutrients are rapid enough to sustain the rapid nutrient uptake by roots. In addition to nutrient uptake, roots play an indispensable role in anchoring the plant body to the ground that mycorrhizae are unable to do, and this role becomes more important when plants grow larger under nutrient-enriched conditions. It has been suggested that gramineous plants have evolved finer roots to obtain a larger surface area per unit carbon investment, that is, toward less dependency to mycorrhizae (Ma et al., 2018). Our findings suggest, however, that maize still maintains balanced strategies, that is, the fine-tuning system of the two nutrient uptake pathways, indicating the importance of the mycorrhizal pathway in foraging, even in the genotypes developed for modern agriculture.

## Conclusion

Our cross-ecosystem transcriptomics approach provides new insights into understanding gene-environment interactions in plant foraging strategies by defining the three gene coexpression modules for mycorrhiza formation, PSR, and root development. The constancy of the relative expression levels of module member genes among the genotype site combinations suggests that the genetic modules defined by this approach are robustly regulated across ecosystems and that their regulatory systems are conserved at the species level.

Our findings have important implications for conservation agriculture. The identification of soil and plant factors that drive the foraging modules would enable us to reduce the environmental impacts of agriculture by manipulating the factors, e.g., by balancing fertilizer input, improving soil physical conditions, and inoculating with AM fungi. Moreover, although the genotypic variation in module expression (i.e., differences in absolute expression levels of the modules) has not been investigated in detail in this study, it is of interest to characterize genotypes based on, e.g., capability of acquiring more nutrients from organic fractions and/or *via* the mycorrhizal pathway. For this purpose, eigengenes are applicable as a metric to evaluate genotypic performance in field-grown plants, which would contribute to breeding programs in selecting genotypes for low-input food production.

## Data Availability Statement

The RNA-Seq reads have been deposited in National Center for Biotechnology Information under the accession number PRJNA586746 (mRNA-Seq, 251 samples) and PRJNA604657 (rRNA-seq, 20 samples). The expression data of the maize roots and AM fungal transcript references can be downloaded from: http://lab.agr.hokudai.ac.jp/botagr/rhizo/RhizoCont/Download.html.

## Author Contributions

HM, AK, and TE conceived the project. AK and TE designed the field surveys. YS, HM, AK, and TE collected the samples. YS, HM, and AK extracted the RNA and analyzed the plant and soil samples under the supervision of TE. YS performed the quality assessment and mapping of the sequenced reads. YS and TE analyzed the data and interpreted the results. YS and TE wrote the manuscript with input from all the authors, and all the authors discussed the manuscript.

## Conflict of Interest

AK was employed by Sumitomo Chemical, Co., Ltd. The remaining authors declare that the research was conducted in the absence of any commercial or financial relationships that could be construed as a potential conflict of interest.

## Publisher’s Note

All claims expressed in this article are solely those of the authors and do not necessarily represent those of their affiliated organizations, or those of the publisher, the editors and the reviewers. Any product that may be evaluated in this article, or claim that may be made by its manufacturer, is not guaranteed or endorsed by the publisher.

## Footnotes

1
ftp://ftp.ensemblgenomes.org/pub/plants/release-36/fasta/zea_mays/dna/

2
http://CRAN.R-project.org/package=vegan

3
https://transdecoder.github.io
