# Supplementary material for: Plant Foraging Strategies Driven by Distinct Genetic Modules: Cross-Ecosystem Transcriptomics Approach
Source: Front Plant Sci. 2022 Jul 4;13:903539. doi: 10.3389/fpls.2022.903539 (PMC9290524; doi:10.3389/fpls.2022.903539)
Supplement: Supplementary file 1 [file Data_Sheet_1.zip › Supplementary Figures.pdf]

Supplementary Material

Supplementary Figures

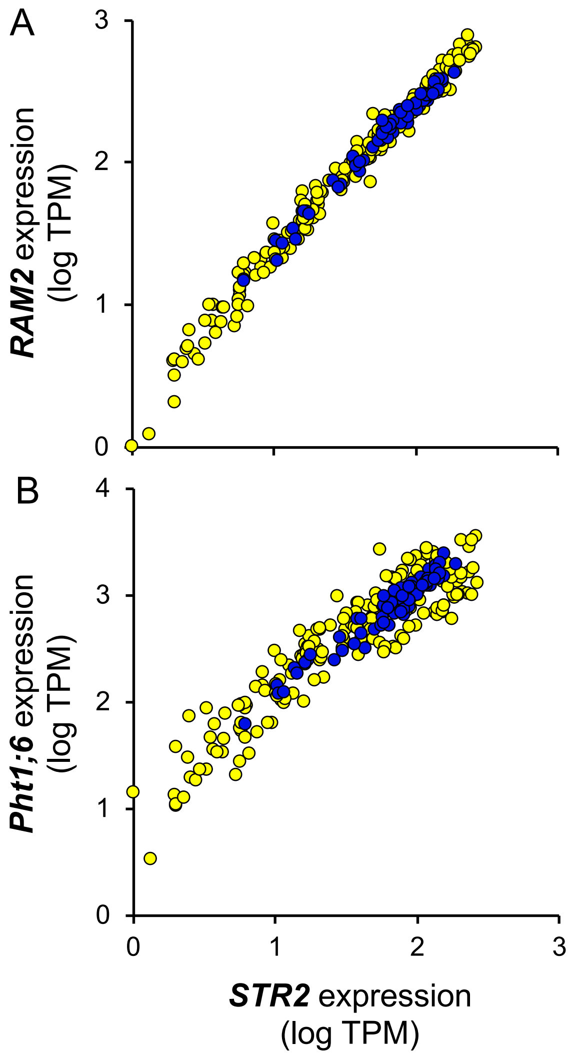

Supplementary figure 1 Correlations of the expression levels of *STR2* with those of *RAM2* (A) and *Pht1;6* (B) that are essential for arbuscule development and functioning. Correlation coefficients between *STR2* and *RAM2* and between *STR2* and *Pht1;6* are 0.992 and 0.939, respectively ($P < 0.001$). Blue and yellow dots represent the samples collected from USA and Japan, respectively.

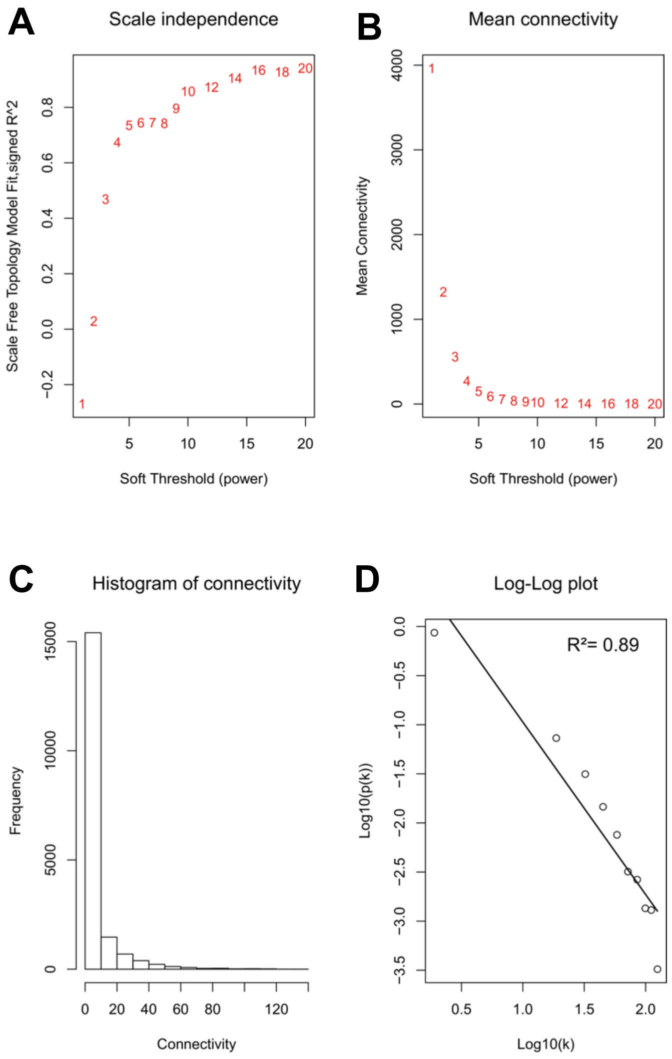

Supplementary figure 2 Evaluation of weighted coexpression network properties in maize roots. **A**, Scale-free fit index (y-axis) as a function of soft-threshold power (x-axis). **B**, Mean connectivity (degree, y-axis) as a function of soft-threshold power (x-axis). **C**, Frequency distribution of network connectivities. **D** Log-log plot of edges [log10(k)] versus probability of a node having k edges [P(k)].

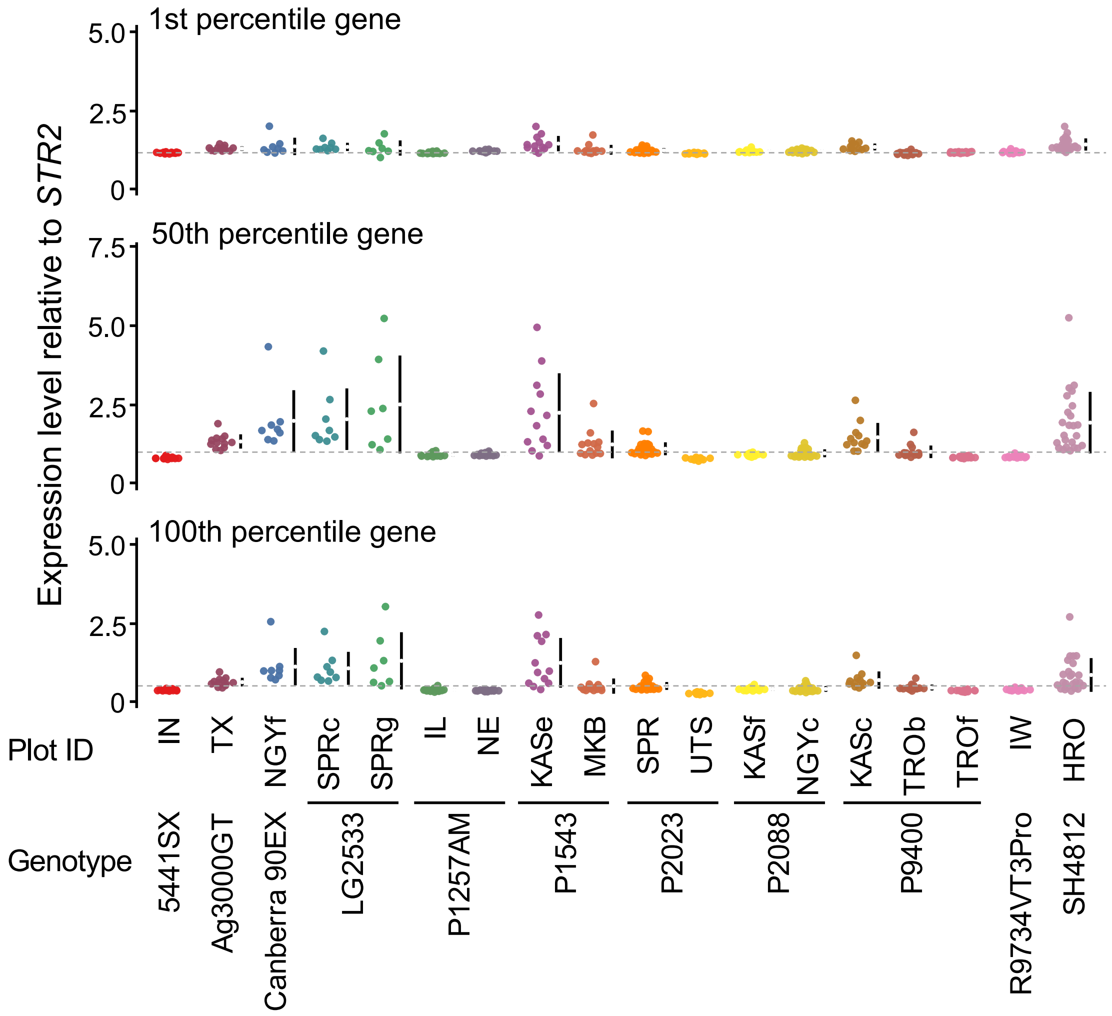

Supplementary figure 3 Expression levels of the genes that showed 1st (Zm00001d033915), 50th (Zm00001d033002), and 100th (Zm00001d032267) percentile connectivity in the mycorrhizal module relative to those of *STR2* (Zm00001d043722) that showed the highest-connectivity in individual genotype-site combinations. Dotted lines indicate average values. Plot IDs are listed in Table S4. Vertical bars indicate 95% confidence intervals.

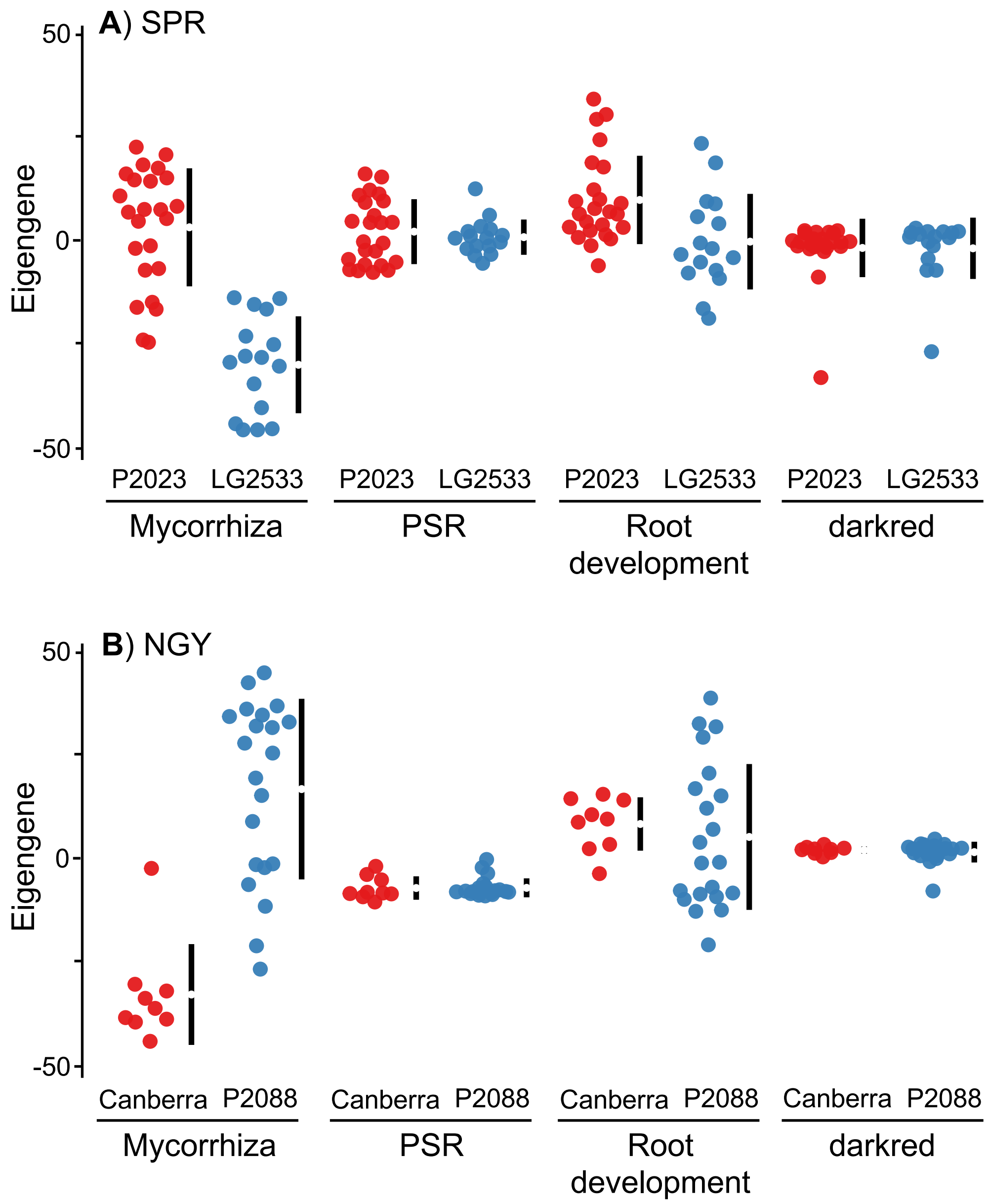

Supplementary figure 4 Absolute expression levels (eigengenes) of the mycorrhizal, PSR, root development, and darkred modules in the genotypes P2023 and LG2533 grown in Sapporo site (SPR), respectively, and in Canbera 90EX and P2088 grown in Nagoya site (NGY), respectively. Vertical bars indicate 95% confidence intervals.

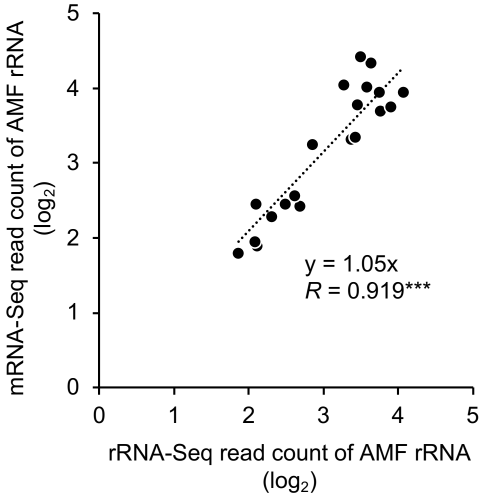

Supplementary figure 5 Correlation analysis between the AM fungal rRNA read numbers obtained by mRNA-Seq and rRNA-Seq. Twenty samples were randomly chosen from the 251 RNA extracts and subjected to rRNA-Seq (i.e., RNA-Seq without purification of mRNA). The single-end 75-base sequence reads obtained by the two methods were assigned to maize LSU rRNA and 524 AM fungal operational taxonomic units by Blastn searches. The read counts were normalized to unit nt length, and total AM fungal read counts in the individual samples were standardized by 10^5 plant rRNA reads and transformed to logarithmic (log_2) values.

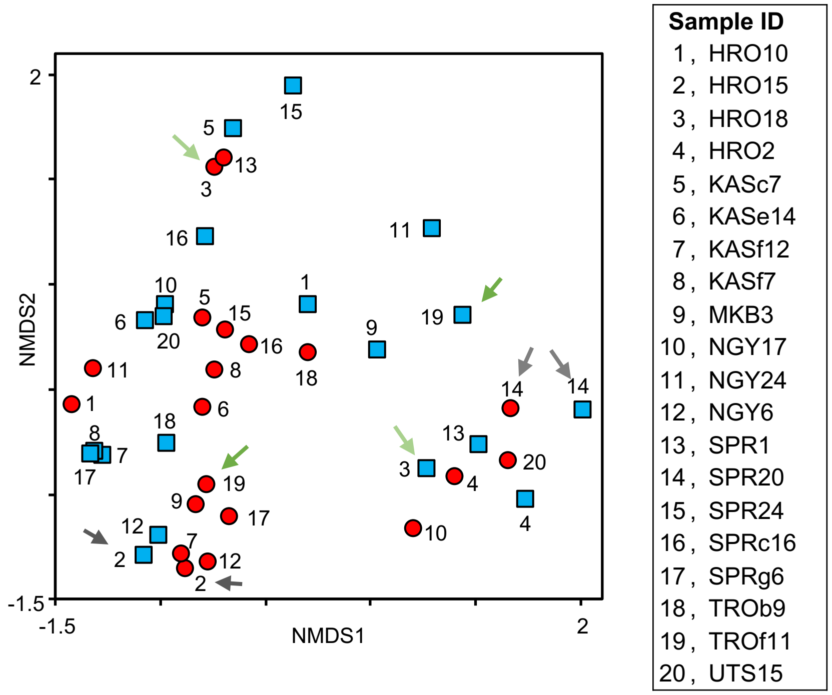

Supplementary figure 6 Non-metric multidimensional scaling (NMDS) on the AM fungal communities in randomly selected 20 samples revealed by mRNA-Seq (red circles) and rRNA-Seq (blue squares) using Bray-Curtis dissimilarity index as a metric. The sequence reads were normalized on the basis of plant rRNA read numbers (Supplementary table 9 for mRNA-Seq and table 10 for rRNA-Seq) and transformed to logarithmic values (log_2) before analysis. Gray and green arrows indicate examples that showed small and large differences, respectively, in the index within the same samples.

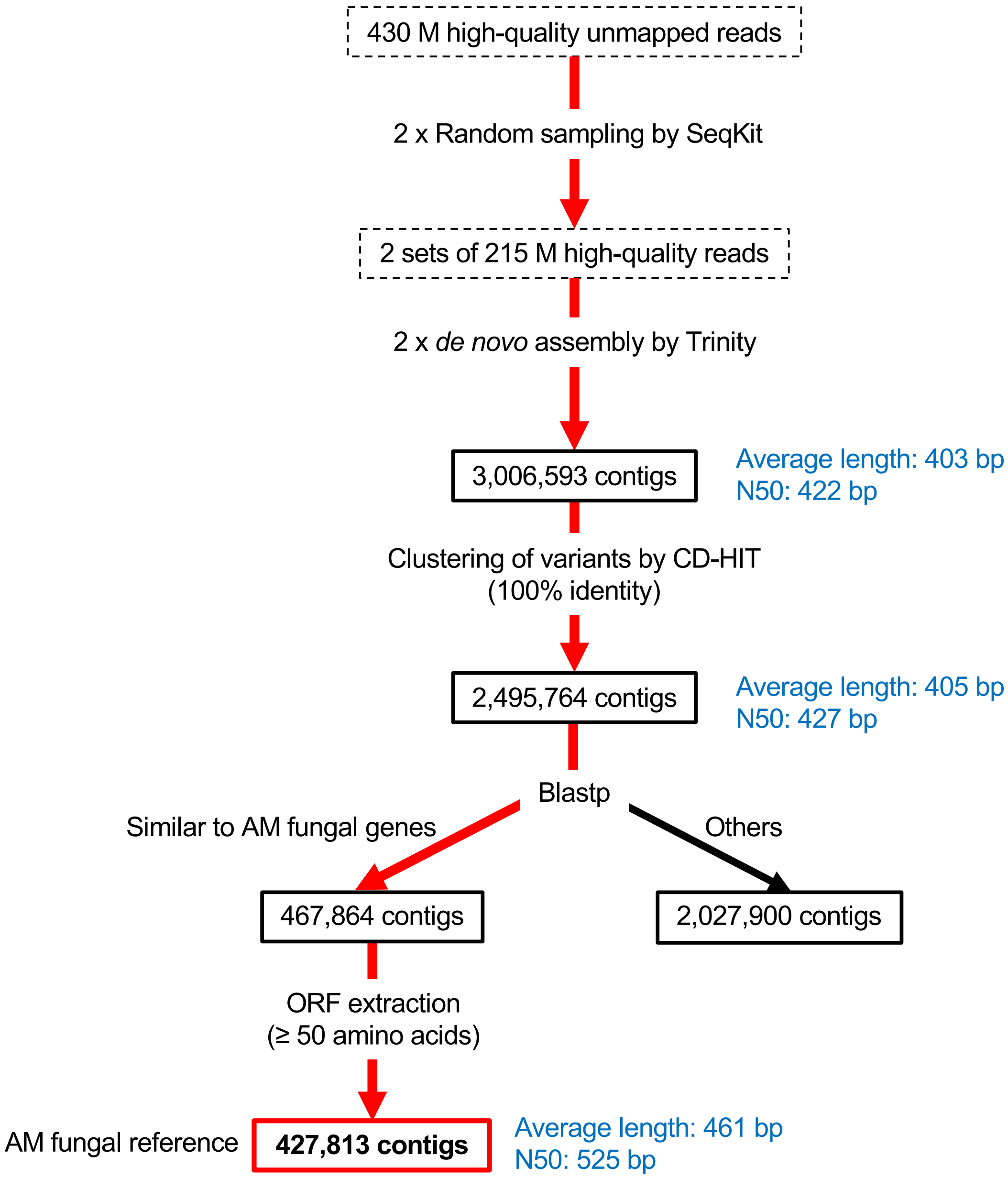

Supplementary figure 7 Procedure for the construction of AM fungal transcript references from the unmapped reads.

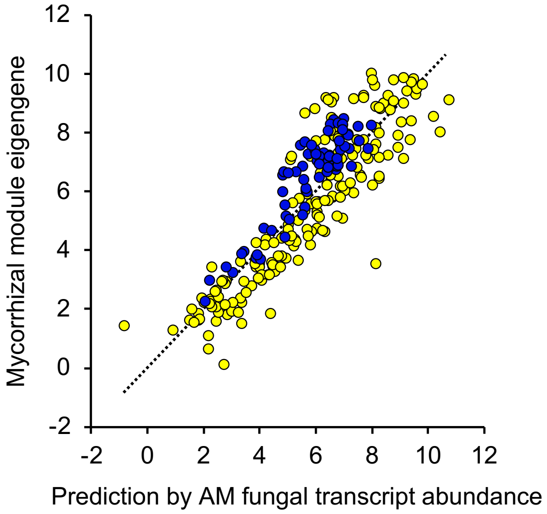

Supplementary figure 8 Interpretation of mycorrhizal module eigengenes by multiple regression analysis with the transcript abundance of the AM fungal *SYG1-1*, *PHO91* and rRNA of which correlation coefficients are 0.150, 0.233, and 0.875, respectively, with a determination coefficient (R^2) of 0.782 ($P < 0.001$). The transcript abundances were total numbers of the reads that showed similarity to the known AM fungal genes *SYG1-1* and *PHO91* and those of the reads that were assigned to AM fungal LSU rDNA operational taxonomic units within each sample. Blue and yellow dots represent the samples collected from USA and Japan, respectively.

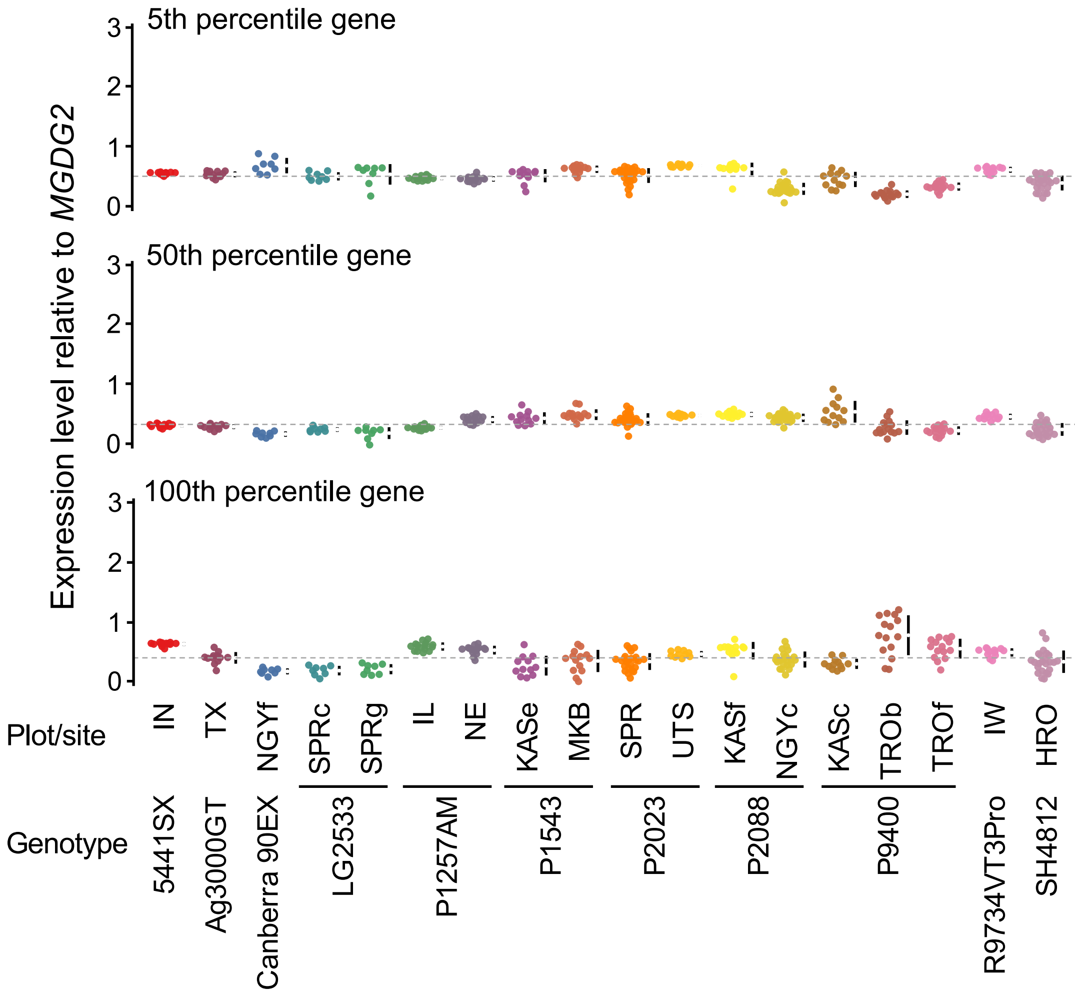

Supplementary figure 9 Expression levels of the genes that showed 5th (Zm00001d026156), 50th (Zm00001d043681), and 100th (Zm00001d020985) percentile connectivity in the PSR module relative to those of *MGDG2* (monogalactosyldiacylglycerol synthase 2, Zm00001d031428) that showed the highest-connectivity in individual genotype-site combinations. Dotted lines indicate average values. Plot IDs are listed in Table S4. Vertical bars indicate 95% confidence intervals.

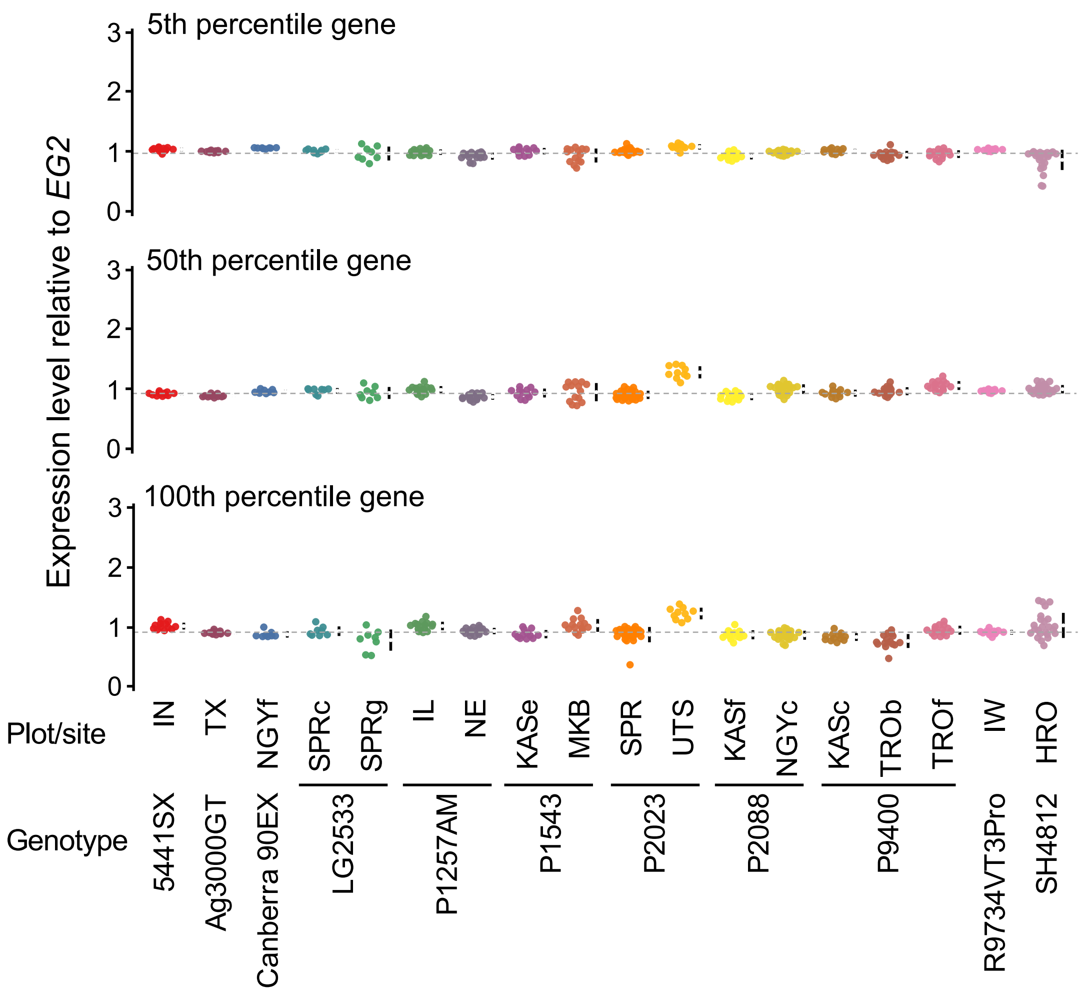

Supplementary figure 10 Expression levels of the genes that showed 5th (Zm00001d010976), 50th (Zm00001d042276), and 100th (Zm00001d006756) percentile connectivity in the root development module relative to *EG2* (endoglucanase 2, Zm00001d021304) that showed the highest connectivity in individual genotype-site combinations. Dotted lines indicate average values. Plot IDs are listed in Table S4. Vertical bars indicate 95% confidence intervals.

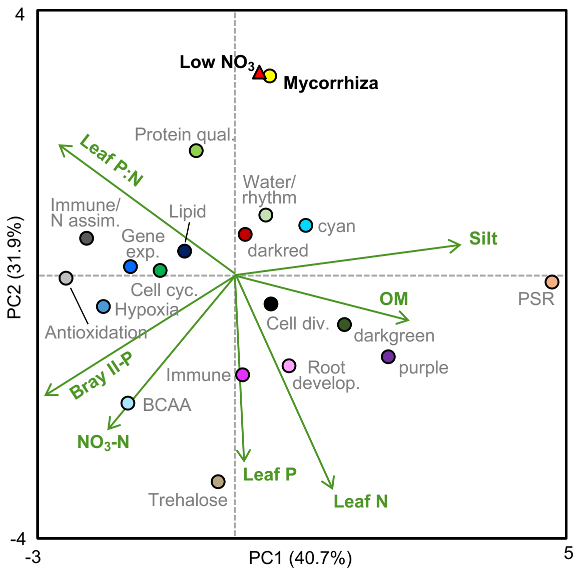

Supplementary figure 11 PCA biplot of module-factor correlations. The plot was drawn based on the correlation coefficients obtained by pairwise correlation analysis between the soil/plant factors and module eigengenes (Table S14), in which factors were selected by taking into account multicollinearity. The module names (functions) and colors are listed in Table 1, and the mycorrhizal and low-NO_3 responsive modules were indicated with bold black letters.

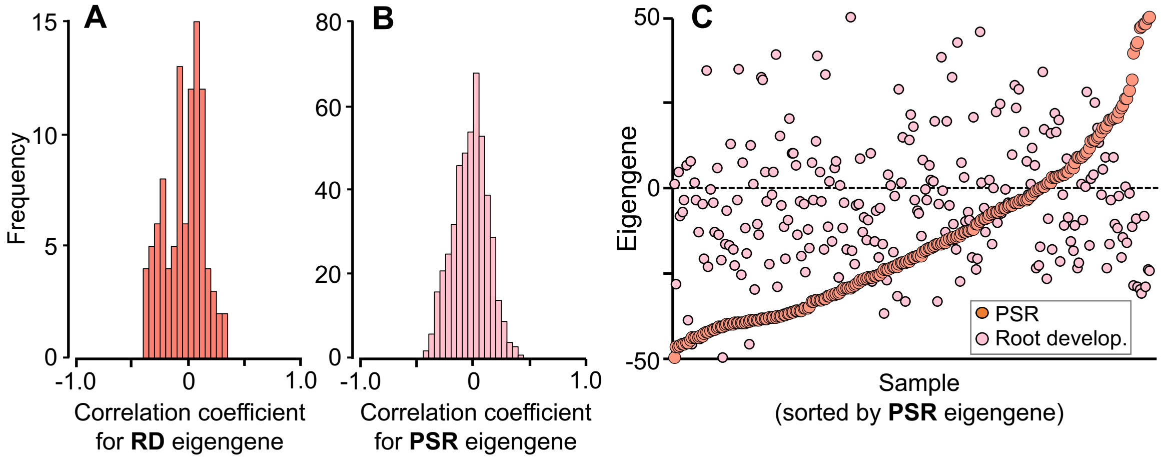

Supplementary figure 12 Interplays between the PSR and root development (RD) modules. Frequency distributions of correlation coefficients of the PSR module genes with the root development module eigengenes (**A**), and those of the root development module genes with the PSR module eigengenes (**B**). **C**, Scatter plot of the eigengenes of the PSR (salmon) and root development (pink) modules of the 251 samples, in which the samples were sorted by the order of PSR module eigengenes. The data were extracted from Supplementary table 6, and all the eigengenes were standardized between -50 (minimum value) and +50 (maximum value) for plotting.
